# Access to civil justice as a social determinant of health: a legal epidemiological cross-sectional study

**DOI:** 10.1186/s12939-024-02205-4

**Published:** 2024-06-14

**Authors:** Eddy Hin Chung Fung, Dong Dong

**Affiliations:** 1 Faculty of Law, The Chinese University of Hong Kong, Hong Kong, China; 2 JC School of Public Health and Primary Care, The Chinese University of Hong Kong, Hong Kong, China; 3 Centre for Health Systems and Policy Research, The Chinese University of Hong Kong, Hong Kong, China

**Keywords:** Access to justice, Civil justice, Social determinants of health, Hong Kong, Law for health, Medical-legal partnership, Health-justice partnership

## Abstract

**Background:**

Although it is widely acknowledged that access to civil justice (ATJ) is a key social determinant of health (SDOH), the existing literature lacks empirical evidence supporting ATJ as a SDOH for specific dimensions of health.

**Methods:**

A legal epidemiological, cross-sectional, postal survey was conducted on *n* = 908 randomly sampled participants in Hong Kong in March 2023. Data collected were perceptions of the civil justice system, health, and sociodemographics. Perceived ATJ was assessed using a modified version of the Inaccessibility of Justice scale (IOJ) and Perceived Inequality of Justice scale (PIJ), i.e. the “modified IOJ-PIJ”, consisting of 12 of the original 13 items from both scales divided into two subdomains: “procedural fairness”, and “outcome neutrality”. For health data, quality of life was assessed using the Hong Kong version of the Abbreviated World Health Organization Quality of Life questionnaire (WHOQOL-BREF(HK)), psychological distress (including symptoms of anxiety and depression) was assessed using the four-Item Patient Health Questionnaire (PHQ-4), and having comorbidities was assessed using Sangha’s Self-Administered Comorbidity Questionnaire (SCQ). Structural equation modelling (SEM) was used to investigate the relationships between perceived ATJ and the measured health outcomes.

**Results:**

SEM demonstrated that both subdomains for ATJ had significantly negative associations (B < 0; *p* < 0.05) with all quality-of-life subdomains, except for between outcome neutrality with social relationships; both subdomains for ATJ had significantly positive association (B > 0; *p* < 0.05) with both anxiety and depression; and, after adjusting for age, only “procedural fairness” had significantly positive association (B > 0; *p* < 0.05) with having comorbidities.

**Conclusion:**

This study provided empirical evidence that ATJ is a SDOH for specific dimensions of health. The results of this study encourage laws, policies, and initiatives aimed at improving ATJ, as well as collaborative efforts from the legal and health sectors through health-justice partnerships, and from the broader community, to safeguard and promote public health by strengthening ATJ.

**Supplementary Information:**

The online version contains supplementary material available at 10.1186/s12939-024-02205-4.

## Introduction

There is increasing acknowledgement that access to civil justice (ATJ) is an important social determinant of health (SDOH) [1–6]. However, the existing literature that links ATJ as a SDOH lacks empirical evidence to support the relationship between ATJ and health outcomes. Despite legal issues being embedded in most SDOH, law has been largely invisible in SDOH discourse, interventions, and research [4]. Indeed, public health law *research* remains a “nascent field” [7], in particular, the relationship between ATJ and health is underexplored [2, 5].

Yet, ATJ is especially important to health since health and justice problems disproportionately affect disadvantaged or vulnerable groups, who are marginalised based on, *inter alia*, their race, gender, class, disability, or sexual identity, perpetuating their social exclusion [2, 5, 8]. Therefore, inaccessibility to justice represents a point of significant health inequity.

To create the conditions for healthy populations, there is a need for evidence-based public health laws that are equity promoting, engages with sectors beyond health, and are supported by good governance [9]. Empirical evidence is important for public health law practice and scholarship since it justifies regulatory action and helps identify the most desirable policies that are effective and consistent with human rights or other legal standards [10]. Although not all law is or can be “evidence based”, even in public health, “[t]he responsible use of law as a tool for improving public health requires a commitment to the pursuit and consideration of scientific evidence when possible” [10].

To provide empirical evidence to support ATJ as a SDOH which can drive evidence-based laws, policies, and initiatives to improve ATJ, this study aims to investigate the association between ATJ and certain health outcomes including quality of life, psychological distress (including symptoms of anxiety and depression), and the presence of medical conditions (i.e., comorbidities).

### ATJ

The Organisation for Economic Co-operation and Development (OECD) defines ATJ as “broadly concerned with the ability of people to obtain just resolution of justiciable problems and enforce their rights, in compliance with human rights standards, if necessary, through impartial formal or informal institutions of justice and with appropriate legal support” (citations omitted) [11].

Although access to the *civil* justice and *criminal* justice systems are often conflated with one another [12], this study is squarely concerned with access to the former, which is “access to tools necessary for the effective resolution of legal disputes outside of the criminal justice system” [2]. Examples of legal problems that are dealt with by the civil justice system are employment disputes over the unreasonable termination of an employment contract, compensation for personal injuries from the negligence of others, disputes over how assets are distributed in a divorce, and disputes between landlord and tenant for rent in arrears.

The scope of ATJ may be defined narrowly or broadly. Historically, ATJ has been narrowly defined to include access to lawyers and the ability for individuals to address their legal needs through courts and tribunals. Today, a broader definition of ATJ is widely adopted which includes access to other legal practitioners or institutions such as paralegals and community legal clinics, access to alternative dispute resolution outside the courtrooms, the ease of navigating the legal system, the feasibility of self-representation, the sociocultural sensitivity of justice providers, the responsiveness of the legal system to the needs of marginalised groups, as well as the ability to address systemic barriers to achieving justice through laws, policies, and initiatives [2]. This study adopts the broad definition of ATJ.

Indeed, ATJ is important to a well-functioning justice system since without it, “[s]ubstantive law such as contract law, corporate law and securities law would be dead letter as mere law on the books” [13]. ATJ is also understood as the “bedrock principle” undergirding human rights [14], without which rights are “abstract and meaningless” [2].

ATJ should not be confused with legal needs, as they are separate constructs. The OECD notes that “[l]egal needs surveys are distinct from other forms of access to justice assessment survey” [11]. Additionally, Pleasence P and Balmer NJ (2018) found that experiencing civil legal need is not significantly associated with perceived ATJ [12]. Furthermore, Schram A, Boyd-Caine T, et al. (2021) notes [6]:

“**Enabling access to justice is about more than just resolving legal problems**; it is about reducing social inequities that produce health inequities, breaking vicious cycles that create and compound poverty, undermine socioeconomic development, and contribute to broader social inequality” (emphasis added, internal quotation marks and citations omitted).

### SDOH

Today, it is widely recognised that health and wellbeing are affected by both the individual biological and genetic factors, as well as social and environmental circumstances. The SDOH framework by the World Health Organisation (WHO) represents a shift from the biomedical model to the social model of health, it explains that our health is a function of the wider conditions in which people are “born, grow, live, work, and age” [15]. Understood this way, SDOH are the “causes of the causes” to health [16].

The wider conditions that affect the health outcomes of individuals and populations include, *inter alia*, political, cultural, and socioeconomic factors [15]. When SDOH, including money, power, and resources, are unfairly distributed among the population at the global, national, and/or local levels, *health inequities* arise which refers to the “differences which are *unnecessary* and *avoidable*, but in addition are considered *unfair* and *unjust*” (emphasis in original) [17]. Since SDOH influences between 45 and 57% of our health [18], addressing SDOH is understood as a cost-effective way of promoting population health [19–22].

### ATJ as a SDOH

There is increasing acknowledgement that ATJ is a SDOH [1, 2, 4–6]. The scope of ATJ has traditionally been limited to the legal community, thus the burden to address the issue has been placed on members of the legal community including lawyers and law schools [5]. However, the scope of ATJ continues to evolve, there are ongoing efforts to broaden the scope beyond the legal domain [5]. According to the OECD, “This is beginning to shift. Numerous countries have begun to bring broader planning for access to justice into their development and strategic plans” [11].

An area into which the scope of ATJ has been expanding is the health field. Nobleman RL (2014) argues that ATJ should be explored as a “discrete” SDOH [2], and that it is time to expand our view of SDOH to include ATJ [2]. ATJ, as a SDOH, can therefore be framed as the “cause of causes”, falling within the WHO’s description of SDOH as the wider circumstances in which people are “born, grow, live, work, and age” [5, 15]. This is supported by Jassar S (2022) noting that access to legal services can address the underlying causes of health conditions in marginalized communities, leading to improved health outcomes and contributing to lasting health equity [5].

When people have ATJ to deal with the underlying causes of their ill health, it can serve as a type of preventative healthcare [1, 2, 9, 23]. ATJ is therefore a key component of the right to health [24]. To put in stronger terms, “not recognizing that a major factor underlying health disparity is access to justice. Even the most progressive [SDOH] model fails to address access to justice or law as a whole” [5]. Therefore, framing ATJ as a SDOH is a necessary first step to establish a comprehensive and interdisciplinary approach to promote health equity [5].

Having civil legal problems is associated with negative health outcomes. Previous study in England and Wales by Pleasence P, Balmer NJ, et al. (2008) found that those reporting to have a civil legal problem were significantly more likely to report long-term illness/disability [25]. In that study, among 18 types of civil legal problems, 10 were significantly associated with the reporting of long-term illness/disability [25]. That study provides a good summary of the relationship between having civil legal problems and adverse health outcomes [25]:

“The relationship between some types of civil-law problem and ill health is readily apparent. Domestic violence and negligent accidents can result in serious physical and psychological injury, even death (or miscarriage). They can also follow on from physical or mental incapacity. Evidently, also, nonviolent ‘family’ problems, including divorce, can cause and be brought about by long-term psychological health problems. Further, associations have been found between ill health and poor and overcrowded housing, homelessness, debt, discrimination, and problems with employment” (citations omitted).

However, where previous studies have used empirical evidence from legal needs surveys to support the argument that ATJ is a SDOH, this may be lacking since, as discussed previously, legal needs surveys are different from surveys for ATJ [11].

### Research gap

Existing literature relating ATJ as a SDOH is *argumentative*. Where empirical evidence is used to support the link between ATJ and health, empirical studies from legal needs surveys are used to indirectly support the relationship [1, 2, 4–6]. However, as discussed previously, legal needs surveys are not the same as ATJ surveys [11]. It is worth noting at this point that there is no formal process of identifying a factor as a SDOH, “[s]ocial determinants of health are generally recognised when diffuse health research around the world builds up evidence that a certain social factor has an impact on population health” [2]. Furthermore, due to the diversity of civil justice problems and their various effects on the lives of people involved, existing literature on ATJ as a SDOH provides an imprecise description of the impacts ATJ has on health and does not go into detail the effect ATJ has on *specific dimensions* of health, such as the physical, psychological, or social dimensions of health [2]. The lacuna in the existing literature is, therefore, the lack of *empirical* research supporting ATJ as a SDOH, and the lack of such research specifying the effect ATJ has on the *specific dimensions* of health.

### Aim of this study

To fill the research gap previously identified, this study aims to investigate the association between ATJ and certain health outcomes including quality of life, psychological distress (including symptoms of anxiety and depression), and the presence of medical conditions (i.e., comorbidities).

## Method

### Sampling and recruitment

Sampling and recruitment took place in March 2023. This study utilised a legal epidemiological, population-based, cross-sectional, postal survey study design with random sampling. The sampling list consisted of 10,079 randomly selected addresses obtained from the frame of quarters maintained by the Census and Statistics Department of Hong Kong. This is the most up-to-date, complete and authoritative sampling frame available in Hong Kong [26].

Letters were mailed to all addresses in the sampling list. Each letter contained, in Chinese and English, (i) an invitation to participate in this study; (ii) a unique QR code to an online version of the survey; (iii) a paper-based version of the survey with a unique identifier (Supplementary Material 1); (iv) a pre-paid envelope for the participant to mail the paper-based survey back to the authors; and (v) instructions on mailing the paper-based survey.

Participants were eligible to participate if they were at least 18 years old and living in Hong Kong. If the address had more than one resident, only the eligible resident whose next birthday was closest to the date of the invitation letter (i.e., 01 March 2023) was invited to participate.

To participate in this study, participants could either (i) scan the QR code to complete the survey online, or (ii) complete the paper-based survey and mail it to the authors. To avoid sample double-counting due to the completion of both the online and paper-based surveys from the same letter, the unique QR code was matched to the unique identifier on the paper-based survey, allowing for the detection of double-counting.

Written informed consent was obtained from all participants. For participants who completed the online survey, written informed consent was obtained by participants selecting “I agree to participate in this survey” and providing their electronic signature, only by doing so could the participant proceed with the survey. For participants who completed the paper-based survey, written informed consent was obtained by participants signing the informed consent form.

The first 100 participants who validly completed the survey were eligible to receive 200HKD (≈ 25.6USD) cash reimbursement. A question at the end of the survey asked for the participants’ contact information so the authors could arrange the reimbursement.

### Data collection

Online and paper-based data collection were used complementarily. This allowed smartphones or other devices to be used to conveniently complete the survey, yet those technologically challenged could still participate. Participants took around 20 min to complete the survey. The online survey was made using Qualtrics. The native “force response” function on Qualtrics was used such that the online survey could not be completed if any items were missed.

If the participant completed the paper-based survey, it could be mailed back to the authors using the prepaid envelope at no cost to the participant. Where the participant wished to participate but was unable to complete the online or paper-based survey, he/she could contact the authors (via the contact information left on every item in the letter) and the authors would arrange face-to-face data collection subject to COVID-19 control measures in place at the time.

All items in the letters were bilingually available in Traditional Chinese and English. The online and paper-based surveys collected the same data on (i) perceived ATJ, (ii) health, and (iii) sociodemographics. No personally identifiable information was collected.

### Perceived ATJ

Items from the Inaccessibility of Justice scale (IOJ) scale and Perceived Inequality of Justice scale (PIJ), originally developed by Pleasence P and Balmer NJ (2018), were administered to measure perceived ATJ. Prior to this study, a validation study was conducted to validate the Traditional Chinese versions of the IOJ and PIJ for their subsequent use in this study [27]. Based on the results of the prior validation study, it was recommended that the “modified IOJ-PIJ” should be used to represent perceived ATJ. The modified IOJ-PIJ measures perceived ATJ using 12 of the original 13 items from the IOJ and PIJ divided into two subdomains: (i) “procedural fairness”, and (ii) “outcome neutrality”. Each item of the modified IOJ-PIJ collects responses on a four-point Likert Scale (“strongly agree” to “strongly disagree”). Positively worded items scored on a Likert scale from 0 (strongly agree) to 3 (strongly disagree). Negatively worded items were scored in a mirrored reverse manner, except for one item (IOJ5) which scored from 0 (strongly disagree and mainly disagree) to 2 (strongly agree). A higher “modified IOJ-PIJ” score represents higher perceived inaccessibility of justice. The same is true for its two subdomains: a higher “procedural fairness” score represents higher perceived procedural unfairness, while a higher “outcome neutrality” score represents higher perceived outcome non-neutrality. The modified IOJ-PIJ was previously assessed to have acceptable factorial validity (root mean square error of approximation = 0.053; standardized root mean square residual = 0.058; comparative fit index = 0.944; Tucker-Lewis index = 0.930) and internal reliability (Cronbach’s α = 0.708) [27].

### Health

Instruments for collecting health data were: (i) Hong Kong version of the Abbreviated World Health Organization Quality of Life questionnaire (WHOQOL-BREF(HK)); (ii) Four-Item Patient Health Questionnaire (PHQ-4); and (iii) Sangha’s Self-Administered Comorbidity Questionnaire (SCQ). Below describes each instrument in order.

The WHOQOL-BREF(HK) is widely used to measure quality of life in Hong Kong [28]. It consists of 28 items (26 from the original WHOQOL-BREF and two locally adapted items). With the exception of two global items that are examined separately [29], the other 26 items cover four subdomains: (i) physical health, (ii) psychological, (iii) social relationships, and (iv) environmental [28, 30]. Each item scored on a Likert scale of 1–5 and the global and subscale scores were calculated with a higher score indicating higher quality of life [29].

The PHQ-4 measured psychological distress and consisted of four items divided into two subscales: (i) two-item generalised anxiety disorder scale (GAD-2), and (ii) two-item patient health questionnaire (PHQ-2) [31]. Each item scored on a Likert scale from 0 (not at all) to 3 (nearly every day). The PHQ-4 score ranged from 0 to 12, and each subscale score ranged from 0 to 6 [31]. The PHQ-4 score was classified as normal (0–2), mild (3–5), moderate (6–8), and severe (9–12) psychological distress. For GAD-2 and PHQ-2, a score ≥ 3 was considered positive for screening for anxiety and depression respectively [31]. The PHQ-4 has been used before in Hong Kong [32, 33].

The SCQ is a comorbidity questionnaire that consists of 15 items: 13 defined medical problems and two optional conditions [34]. A maximum of three points can be scored for each item: one for the presence of the problem, another if he/she receives treatment for it, and another if the problem causes functional limitations (effectively making the SCQ a 45-item scale) [34]. The scores ranged from 0 to 45 with a higher score representing worse comorbidity. The SCQ can be completed by participants without any medical background [34], it has been used in Chinese settings [35–38] and with Chinese-speaking patients [39–41] before.

### Sociodemographics

For sociodemographics, data collected included district (regrouped into three regions), age, sex, sexual orientation, marital status, education level, employment status, monthly household income, household size, birthplace, Hong Kong permanent resident status, ethnicity, and native language.

### Data analysis

Data analyses conducted were descriptive statistics and structural equation modelling (SEM). Statistical significance was indicated by p-value < 0.05. The dataset used in this analysis is provided in Supplementary Material 2.

### Descriptive statistics

Descriptive statistics of the variables were computed using IBM SPSS 26 and presented as frequencies and percentages for categorical variables and mean ± SD for continuous variables.

### Structural equation modelling

SEM is a *confirmatory modelling technique* used to investigate how well a hypothetical model fits the data [42]. The benefit of SEM is that it allows for the simultaneous estimation of multiple equations describing the relationships between manifest (observed) and latent (unobserved) variables under investigation in a single step, rather than separately estimating each part of the model, thereby increasing overall accuracy [42]. Previous studies have applied SEM to investigate the relationship between the empowerment of Muslim women and improved ATJ [43].

**Fig. 1:**
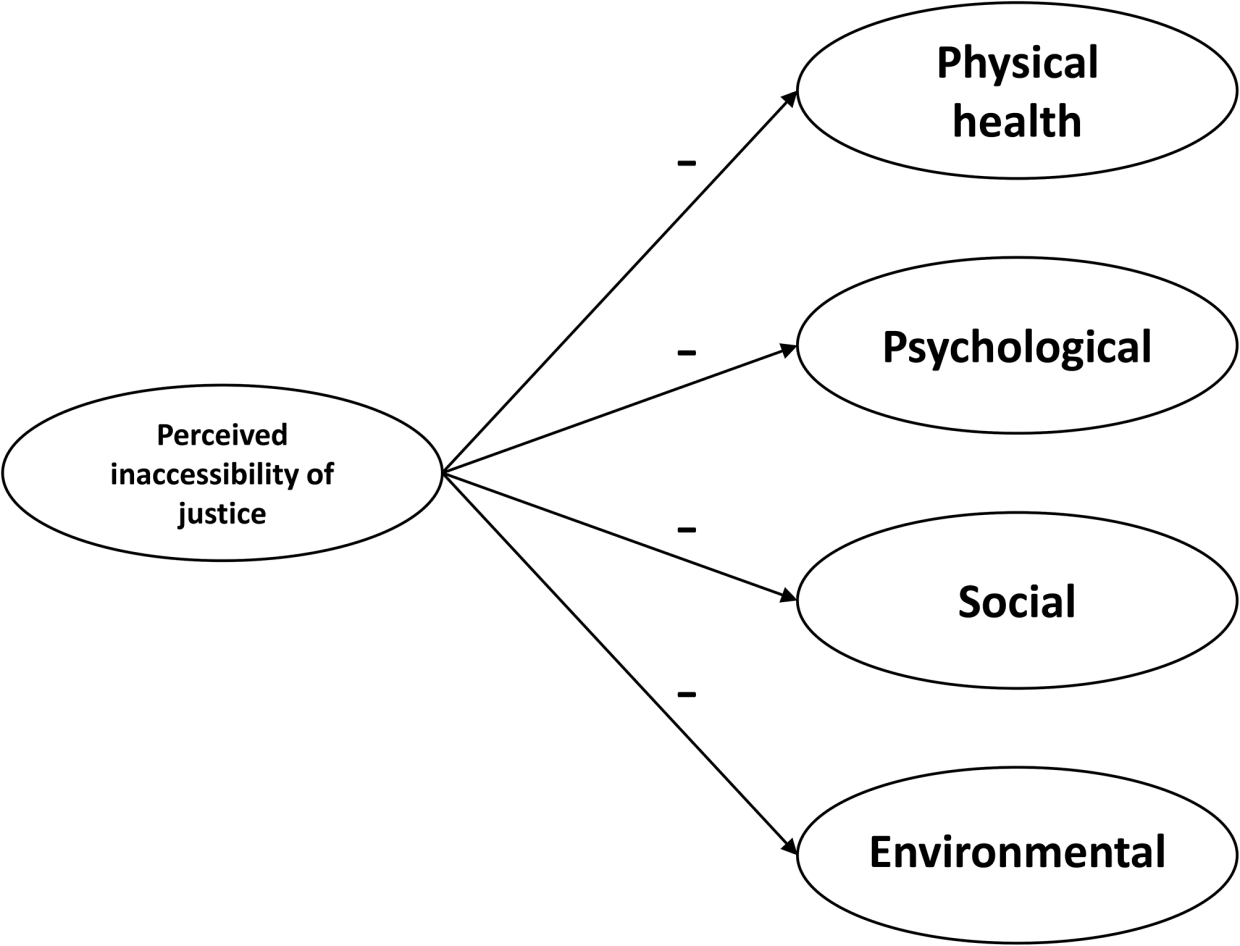
Hypothetical model describing the relationship between access to justice and quality of life

**Fig. 2:**
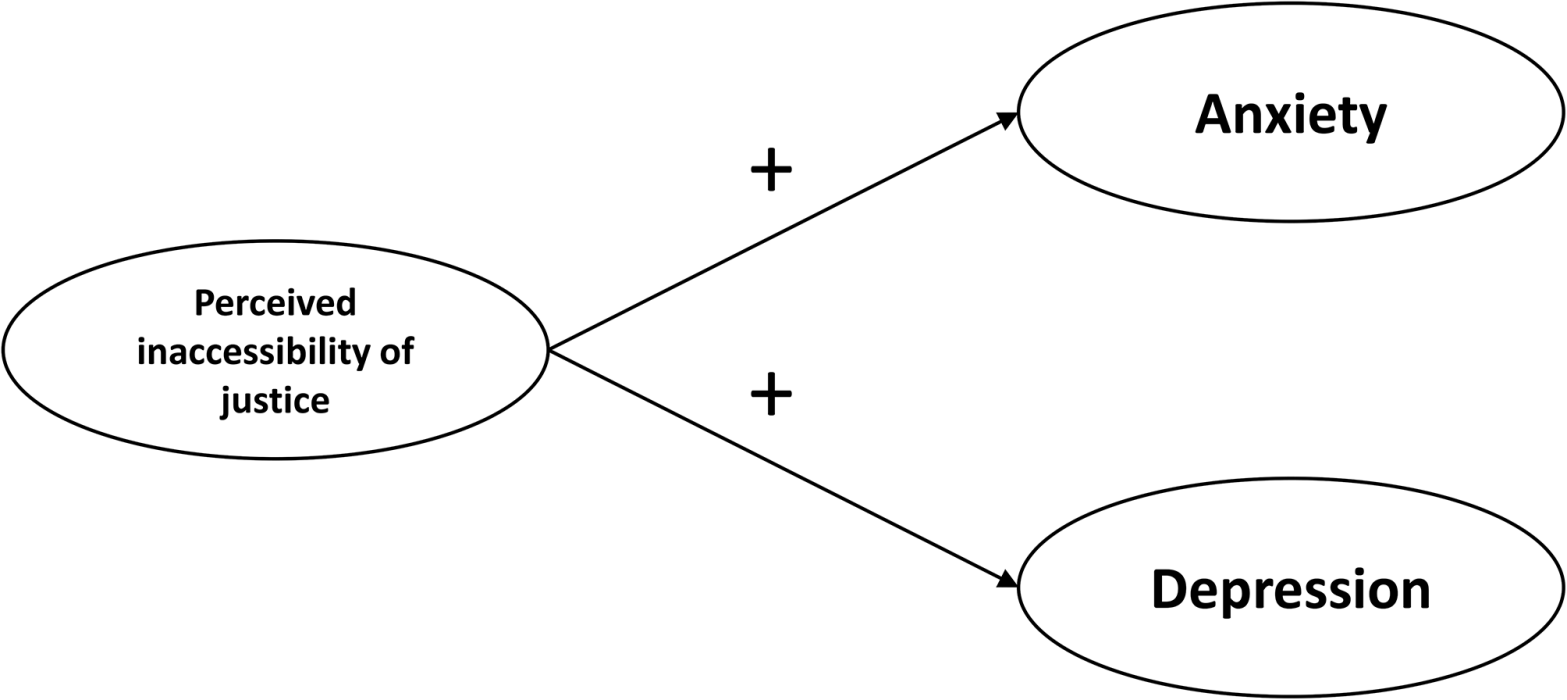
Hypothetical model describing the relationship between perceived inaccessibility of justice and psychological distress

**Fig. 3:**
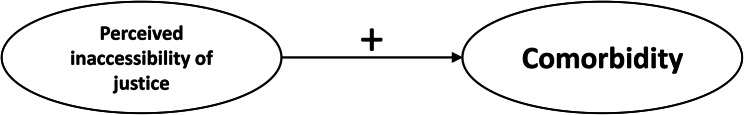
Hypothetical model describing the relationship between perceived inaccessibility of justice and having comorbidities

SEM was conducted using R 4.2.2 with *lavaan* 0.6–14 [44] and *semPlot* 1.1.6 packages [45]. Prior to data collection and analysis, hypothetical models that described the directional relationship between ATJ and specific dimensions of health were specified based on the aims of this study and the existing literature (an overview of the literature was provided in the Introduction above) [42]. Three hypothetical models (collectively, “all three HMs”) were specified, they describe the relationship between:

1. ATJ and quality of life (HM1) (Fig. 1).
2. ATJ and psychological distress (HM2) (Fig. 2); and.
3. ATJ and comorbidities (HM3) (Fig. 3).

SEM was conducted to test the fitness of all three HMs. For all three HMs, the exogenous independent variables were modelled using the modified IOJ-PIJ as latent variable. Furthermore, covariance between exogenous variables and residuals of endogenous variables were modelled. For HM1 and HM2, the endogenous dependent variables were modelled as latent variables using the subdomains for WHOQOL-BREF(HK) and PHQ-4 respectively. For HM3, the endogenous dependent variable was modelled as a manifest variable using the SCQ score, since modelling the SCQ as a latent variable was likely to arbitrarily reduce the model fit indices given that it effectively has 45 items (as discussed previously).

If the structural models for all three HMs did not produce the hypothesised directional relationship between the independent and dependent variables, it may indicate that the model had not accounted for a confounding variable that was causing the spurious association [46]. In this case, the “prior knowledge” approach was adopted whereby the model was adjusted by some sociodemographic variable guided by the scientific literature [47]. Such an approach to covariate selection is ideal for SEM since it avoids models quickly becoming too complex to be identified [48].

The structural models for all three HMs were fit using diagonally weighted least square (DWLS) [49]. Acceptable model fit was indicated by root mean square error of approximation (RMSEA) < 0.08, standardised root mean square residual (SRMR) < 0.06, and comparative fit index (CFI) and Tucker-Lewis index (TLI) > 0.9 [50]. Unstandardised (B) and standardised (β) parameter estimates were presented alongside their 95% confidence interval (95% CI) and standard error of unstandardised estimates (SE B).

### Sample size calculation

The minimum sample size was determined to be 504, calculated based on the rule of thumb of 200 participants more than eight times the number of manifest variables used in the model with the most manifest variables [42], that being HM1 with 38 manifest variables (12 from the modified IOJ-PIJ and 26 from WHOQOL-BREF(HK)).

## Results

### Participant characteristics

By 12 March 2023, the authors mailed 10,079 letters, among which 9,698 (96.2%) were successfully mailed and 381 were returned. The returned letters were expected since some quarters in the sampling list may be unoccupied or changed to non-residential use. Data collection ceased on 13 July 2023. After removing *n* = 10 paper-based survey responses for having already completed the online survey (double-counting), the survey received *n* = 921 responses (response rate = 9.50%, calculated as the number of responses divided by letters successfully mailed), among which *n* = 750 (81.4%) responded by online survey and *n* = 171 (18.6%) by paper-based survey. After excluding responses that did not consent to participate (*n* = 4) and responses with missing variables (*n* = 9), *n* = 908 (98.6%) provided their informed consent and were included in analysis.

Table 1 presents the sociodemographic characteristics of the respondents. Most of the participants lived in the New Territories (50.7%); were between 18 and 44 years old (60.2%); were female (53.9%); identified as heterosexual or straight (85.1%); were married (46.4%); had undergraduate degree or Bachelor’s degree as their highest education level (34.6%); were employed full-time (54.1%); had monthly household income ≤$39,999 (59.6%); had household size ≤ 3 (65.9%); were born in Hong Kong SAR, China (74.4%); had permanent resident status (95.3%); were ethnically Chinese (96.6%); and had Cantonese as their native language (92.6%);

**Table 1:** Sociodemographic characteristics

Characteristic (*n* = 908)	Freq. (%^a^)
Region	
Hong Kong Island	155 (17.1)
Kowloon	293 (32.3)
New Territories	460 (50.7)
Age	
18–19	36 (4.0)
20–24	86 (9.5)
25–29	93 (10.2)
30–34	109 (12.0)
35–39	111 (12.2)
40–44	112 (12.3)
45–49	74 (8.1)
50–54	75 (8.3)
55–59	61 (6.7)
60–64	65 (7.2)
≥65	86 (9.5)
Sex	
Male	388 (42.7)
Female	489 (53.9)
Non-binary / third gender, other, or prefer not to say	31 (3.4)
Sexual orientation	
Heterosexual or straight	773 (85.1)
Homosexual, bisexual, other, or prefer not to say	135 (14.9)
Marital status	
Not yet married or single	370 (40.7)
Married	421 (46.4)
Living as married or cohabitate	22 (2.4)
Widowed	22 (2.4)
Divorced	63 (6.9)
Separated	10 (1.1)
Highest education level	
No formal education	2 (0.2)
Primary or below	17 (1.9)
Lower secondary (forms 1–3)	73 (8.0)
Upper secondary (forms 4–6 or 7)	230 (25.3)
Associate degree or higher diploma	125 (13.8)
Undergraduate degree or Bachelor degree	314 (34.6)
Master’s degree	137 (15.1)
Doctorate	10 (1.1)
Employment status	
Employed full-time	491 (54.1)
Employed part-time	104 (11.5)
Self-employed full-time	40 (4.4)
Self-employed part-time	21 (2.3)
Business owner	13 (1.4)
Homemaker/domestic duties	61 (6.7)
Unemployed looking for work	37 (4.1)
Unemployed not looking for work	17 (1.9)
Retired	91 (10.0)
Student	95 (10.5)
Disabled	7 (0.8)
Monthly household income	
<$10,000	108 (11.9)
$10,000 - $29,999	301 (33.1)
$30,000 - $49,999	231 (25.4)
$50,000 - $69,999	111 (12.2)
$70,000 - $89,999	65 (7.2)
≥$90,000	92 (10.1)
Household size	
1	109 (12.0)
2	237 (26.1)
3	252 (27.8)
4	195 (21.5)
≥5	115 (12.7)
Birthplace	
Hong Kong SAR, China	676 (74.4)
Outside Hong Kong SAR, China	232 (25.6)
Hong Kong permanent resident status	
Yes	865 (95.3)
No	41 (4.5)
Don’t know	2 (0.2)
Ethnicity	
Chinese (mono- or bi-ethnic)	877 (96.6)
Non-Chinese	31 (3.4)
Native language	
Cantonese	841 (92.6)
Putong hua	110 (12.1)
English	71 (7.8)
Other	24 (2.6)

^a^ Percentage frequency may not sum to 100% due to rounding error

### Modified IOJ-PIJ scores

Table 2 presents the mean ± SD for the scores for each item in modified IOJ-PIJ organised according to their subdomain. Note that IOJ4 is not present since it was the item removed based on the analysis from the prior validation study [27]. A higher score represents higher perceived inaccessibility of justice. For all items, the mean scores laid between 1.4 and 2.3 inclusively.

**Table 2:** Item statistics for modified IOJ-PIJ

Subdomain	Item	Item description	Mean ± SD (*n* = 908)
Procedural fairness	IOJ1	Issues like these are usually resolved promptly and efficiently.	1.5 ± 0.7
	IOJ6	The justice system provides good value for money.	1.4 ± 0.7
	IOJ7	For issues like these, people like me can afford help from a lawyer.	1.8 ± 0.7
	IOJ8	Rich people’s lawyers are no better than poor people’s lawyers.	1.9 ± 0.8
	PIJ1	The law always treat both parties fairly, whatever their background, gender, ethnicity or faith.	1.4 ± 0.8
	PIJ4	Courts and tribunals always treat both parties fairly, whatever their background, gender, ethnicity or faith.	1.4 ± 0.7
Outcome neutrality	IOJ2^a, b^	People with less money generally get a worse outcome.	1.8 ± 0.7
	IOJ3^a, b^	For issues like these, law is like a game in which the skilful and resourceful are more likely to get what they want.	2.3 ± 0.6
	IOJ5^a, c^	For issues like these, lawyers are too expensive for most people to use.	1.5 ± 0.6
	IOJ9^a^	Taking a case to court is generally more trouble than it is worth.	1.9 ± 0.6
	PIJ2^a^	Judges have their own agendas separate from the law.	1.6 ± 0.6
	PIJ3^a^	The decisions and actions of courts are influenced by pressure from the press and politicians.	1.8 ± 0.7

^a^ Item with reverse scoring applied

^b^ Item also used in the PIJ

^c^ Scoring is 0 for “strongly disagree” and “mainly disagree”, 1 for “mainly agree”, and 2 for “strongly agree”

### Health scale scores

Table 3 shows the scale scores for the tools that collected data on health outcomes. The mean ± SD score for WHOQOL-BREF(HK) global was 90.1 ± 14.8, and for the subdomains for physical health was 25.1 ± 4.4, psychological was 27.9 ± 5.3, social relationships was 9.9 ± 2.0, and environmental was 27.3 ± 5.3. The PHQ-4 mean score (3.0 ± 2.8) was moderate. The mean score for GAD-2 (1.6 ± 1.5) and PHQ-2 (1.4 ± 1.4) were not over 3. The mean ± SD SCQ score was 1.7 ± 3.0.

**Table 3:** Scale scores for tools that collected data on health outcomes

Scale	Score mean ± SD (*n* = 908)
WHOQOL-BREF(HK) global	90.1 ± 14.8
Physical health	25.1 ± 4.4
Psychological	27.9 ± 5.3
Social relationships	9.9 ± 2.0
Environmental	27.3 ± 5.3
PHQ-4 (psychological distress)	3.0 ± 2.8
GAD-2 (anxiety)	1.6 ± 1.5
PHQ-2 (depression)	1.4 ± 1.4
SCQ score	1.7 ± 3.0

### Relationship between ATJ with specific dimensions of health

Table 4 presents the model fit indices for the structural models for all three HMs and for the age-adjusted structural model for HM3, they provided evidence of good fit (RMSEA < 0.08, SRMR < 0.06, CFI > 0.9, and TLI > 0.9).

**Table 4:** SEM model fit indices

Structural model	RMSEA	SRMR	CFI	TLI
HM1	**0.036**	**0.052**	**0.977**	**0.975**
HM2	**0.040**	**0.049**	**0.963**	**0.954**
HM3	**0.049**	**0.055**	**0.944**	**0.930**
HM3 age-adjusted	**0.048**	**0.054**	**0.940**	**0.926**

Note: Acceptable model fit indices in bold

**Fig. 4:**
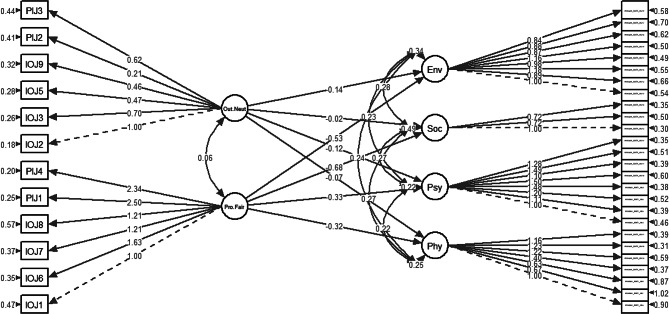
Structural model for HM1. Pro.Fair = Procedural fairness; Out.Neut = Outcome neutrality; Phy = Physical health; Psy = Psychological; Soc = Social relationships; Env = Environmental

Tables 5, 6, 7 and 8 presents the regression parameter estimates for all three HMs and HM3 age-adjusted in *lavaan* model syntax [44]. Tables presenting the detailed structural model and parameter estimates for the same are provided in Supplementary Material 3. In the structural model for HM1 (Fig. 4), both subdomains for ATJ had significantly negative associations (B < 0; *p* < 0.05) with all quality-of-life subdomains as hypothesised, except for between outcome neutrality with social relationships (Table 5).

**Table 5:** Regression parameter estimates for all three HMs

HM1		B (95% CI)	β (95% CI)	SE B	*p*-value
Physical health ∼	Procedural fairness	-0.325 (-0.437 - -0.212)	-0.153 (-0.201 - -0.105)	0.058	< 0.05*
	Outcome neutrality	-0.067 (-0.114 - -0.019)	-0.071 (-0.121 - -0.021)	0.024	< 0.05*
Psychological ∼	Procedural fairness	-0.326 (-0.421 - -0.232)	-0.162 (-0.203 - -0.121)	0.048	< 0.05*
	Outcome neutrality	-0.119 (-0.158 - -0.079)	-0.133 (-0.176 - -0.091)	0.020	< 0.05*
Social relationships ∼	Procedural fairness	-0.680 (-0.896 - -0.465)	-0.228 (-0.293 - -0.162)	0.110	< 0.05*
	Outcome neutrality	-0.021 (-0.110–0.067)	-0.016 (-0.084–0.051)	0.045	0.638
Environmental ∼	Procedural fairness	-0.535 (-0.671 - -0.398)	-0.211 (-0.255 - -0.166)	0.070	< 0.05*
	Outcome neutrality	-0.137 (-0.191 - -0.083)	-0.122 (-0.169 - -0.076)	0.028	< 0.05*

*p < 0.05, **p < 0.01, ***p < 0.001; “.” before the variable name indicates the residual of the variable.

**Fig. 5:**
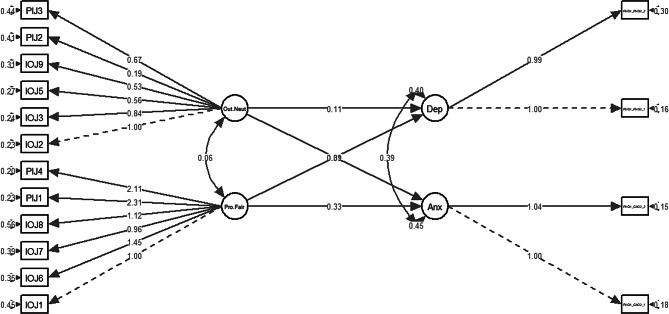
Structural model for HM2. Pro.Fair = Procedural fairness; Out.Neut = Outcome neutrality; Anx = Anxiety; Dep = Depression

In the structural model for HM2 (Fig. 5), both subdomains for ATJ had significantly positive associations (B > 0; *p* < 0.05) with both dimensions of psychological distress (anxiety and depression) as hypothesised (Table 6).

**Table 6:** Regression parameter estimates for HM2

HM2		B (95% CI)	β (95% CI)	SE B	*p*-value
Anxiety ∼	Procedural fairness	0.335 (0.172–0.498)	0.132 (0.070–0.193)	0.083	< 0.05*
	Outcome neutrality	0.091 (0.003–0.179)	0.066 (0.002–0.130)	0.045	< 0.05*
Depression ∼	Procedural fairness	0.313 (0.148–0.478)	0.130 (0.064–0.197)	0.084	< 0.05*
	Outcome neutrality	0.115 (0.025–0.204)	0.089 (0.020–0.157)	0.046	< 0.05*

*p < 0.05, **p < 0.01, ***p < 0.001; “.” before the variable name indicates the residual of the variable.

**Fig. 6:**
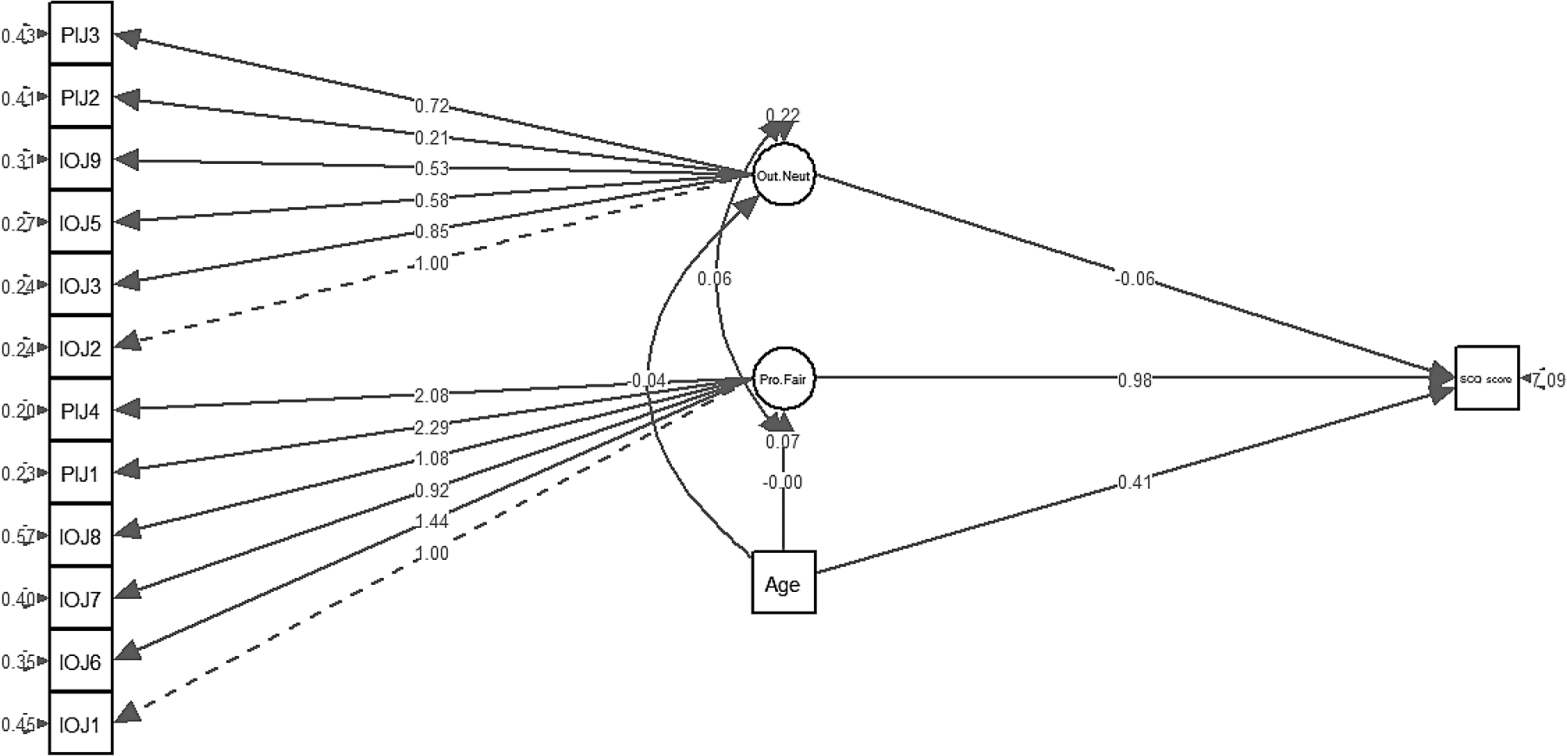
Structural model for HM3. Pro.Fair = Procedural fairness; Out.Neut = Outcome neutrality

In the structural model for HM3, the “procedural fairness” subdomain had significantly positive association (B > 0; *p* < 0.05) with having comorbidities as hypothesised (Table 7). However, the outcome neutrality subdomain had a significantly negative association which was not as hypothesised, this may indicate an unaccounted confounder. Hence, the model was age-adjusted. Age was selected as the sociodemographic variable since increased age was associated with having more comorbidities [51]. After adjusting for age (Fig. 6), age was significantly associated with both subdomains for ATJ, “outcome neutrality” ceased to be significant, and only “procedural fairness” had significantly positive association (B > 0; *p* < 0.05) with having comorbidities as hypothesised (Table 8).

**Table 7:** Regression parameter estimates for HM3

HM3		B (95% CI)	β (95% CI)	SE B	*p*-value
Comorbidity ∼	Procedural fairness	1.369 (0.490–2.248)	0.126 (0.044–0.208)	0.448	< 0.05*
	Outcome neutrality	-0.801 (-1.298 - -0.303)	-0.130 (-0.213 - -0.047)	0.254	< 0.05*

*p < 0.05, **p < 0.01, ***p < 0.001; “.” before the variable name indicates the residual of the variable.

**Table 8:** Regression parameter estimates for HM3 age-adjusted

HM3 age-adjusted		B (95% CI)	β (95% CI)	SE B	*p*-value
Comorbidity ∼	Procedural fairness	0.975 (0.026–1.924)	0.089 (0.002–0.176)	0.484	< 0.05*
	Outcome neutrality	-0.059 (-0.648–0.530)	-0.010 (-0.106–0.087)	0.300	0.844
	Age	0.409 (0.293–0.524)	0.431 (0.288–0.574)	0.059	< 0.05*
Procedural fairness ∼	Age	-0.004 (-0.009–0)	-0.049 (-0.098 - -0.001)	0.002	< 0.05*
Outcome neutrality ∼	Age	-0.037 (-0.047 - -0.028)	-0.240 (-0.294 - -0.186)	0.005	< 0.05*

*p < 0.05, **p < 0.01, ***p < 0.001; “.” before the variable name indicates the residual of the variable.

## Discussion

The purpose of this study was to provide empirical evidence validating the conceptual model that ATJ is a SDOH for specific dimensions of health. The data was gathered directly from a population-based random sample to present ground-level observations of the ATJ situation in Hong Kong. This may inform laws, policies, and initiatives on improving ATJ and health in Hong Kong and other jurisdictions. This section discusses (i) ATJ as a SDOH, (ii) implications of the findings of this study to the current Hong Kong context, and (ii) limitations of this study.

### ATJ as a SDOH

Using SEM, this study confirmed the hypothesis that dimension-specific quality of life (including physical, psychological, social, and environmental), psychological distress (including symptoms of anxiety and depression), and having comorbidities were significantly associated with one or more dimensions of perceived ATJ. This agrees with the literature that argues ATJ to be a key SDOH [1–6].

However, the causal mechanism between ATJ and health outcomes could not be examined with the available data. The causal pathway is likely complicated, consisting of direct and indirect effects, as with most other SDOH [2]. Cataloguing all such possible pathways is impossible [10], yet some remarks should be made here.

A possible, relatively direct pathway is via the allostatic load mechanism [52]. The allostatic load model describes how the cumulative burden of chronic stress can result in adverse health outcomes [52]. Having poor (perceived) ATJ can be a source of psychological distress, contributing to symptoms of mental health conditions including anxiety and depression, which can itself contribute to worsening physical and psychological health. Indeed, previous study by the World Justice Project (WJP) have shown that over 1 in 4 people (29%) reported that they experienced physical or stress-related ill health due to their legal problem [53].

To describe the indirect pathways in which ATJ affects health, the literature employs the language of social exclusion and empowerment to describe how ATJ provides a means to secure other SDOH and address underlying causes of health conditions, thereby breaking cycles of exclusion and improving health [3, 4, 24, 25, 43, 54]. This is emphasised in the SDGs that ATJ, while being central to SDG 16, is “crucial to implementing many of the other SDGs, such as eradicating poverty and hunger (SDG 1 and SDG 2)” [55].

Although this study presents a *unidirectional* relationship between ATJ and health outcomes, whereby ATJ is modelled as affecting health outcomes in a single direction, the literature suggests that the relationship is *bidirectional* instead [2, 4–6, 9]. This is because while ATJ can impact health outcomes, health status can influence ATJ as well – there is a two-way relationship. For example, poorer health, such as having a disability, could make it harder to navigate the legal system or access legal assistance. Future studies should investigate the bidirectional relationship further.

### Implications of the findings of this study to the current Hong Kong context

By way of background, under the policy of “One Country, Two Systems”, Hong Kong adopts the common law legal system which originated from England and, broadly speaking, relies on judicial decisions (i.e., case law) and the doctrine of binding judicial precedents from previously decided court cases (i.e., *stare decisis*) to develop its main body of law [56]. The common law in Hong Kong is a British-colonial leftover by virtue of Article 8 of the Basic Law, Hong Kong’s constitutional document which came into effect on 1 July 1997. This distinguishes Hong Kong’s legal system from the civil law tradition prevalent in the People’s Republic of China (PRC), which originated from Roman Law that later developed in Continental Europe and, broadly speaking, relies on codified statutes as its main source of law [56]. Hong Kong is also the site where the common law has appeared in Chinese for the first time in history [57], contributing to its unique legal heritage.

Issues surrounding access to justice has endured throughout Hong Kong’s history. When Hong Kong was a British colony (1841–1997), accessibility was an issue among the majority Chinese speaking locals in a justice system where its judges were all Europeans and indigenous elites who practiced in English and were primarily interested in the economic interest of the colony, which was often translated as the economic interest of the British colonists and indigenous elites. These, among other institutional limitations that mostly manifested themselves along racial and classist lines, stood as systemic barriers to access to civil and criminal justice. It was only until the enactment of the Official Languages Ordinance (Cap. 5) in 1974 that Chinese became another official language in the region [57].

After the 1997 handover of the British administration of Hong Kong to the PRC, the rights surrounding access to justice were enshrined in Article 35 of the Basic Law and Article 11 of the Hong Kong Bill of Rights Ordinance (Cap. 383). However, these rights were codified in narrow terms, focussing only on access to lawyers, courts, and procedural safeguards, which leaves something to be desired. Today, the use of Chinese is progressing slowly in Hong Kong’s legal system [57], English continues to enjoy superior status as Chinese is considered incapable of expressing common law concepts [57], and calls for improving access to justice particularly among grassroots communities persist ever louder [58]. This study adds to the dearth of ATJ studies in Hong Kong, and advocates for laws, policies, and initiatives to improve ATJ in the region.

Current discourse in Hong Kong on improving access to civil and criminal justice has been entangled in economic, commercial, and realpolitik considerations surrounding the region’s status as an international finance hub, such as: (i) maintaining its leading position as a site for international arbitration; (ii) the role of Hong Kong’s justice system in the Greater Bay Area; and (iii) access to justice in relation to the rule of law, judicial independence, and fundamental rights and freedoms as guaranteed by the Basic Law and undergirded by “One Country, Two Systems”. Health has never been part of that discourse, neither has it been a driving force for improving access to justice in the region. As argued elsewhere in this article, it is imperative to view access to justice as a key SDOH–a healthy city has an accessible justice system.

### Limitations

Due to the cross-sectional design, this study was unable to investigate how health changes with perceived ATJ over time. No cause-and-effect relationship could be concluded. Future longitudinal studies may be conducted to provide more evidence of the temporality between perceived ATJ and health outcomes. Temporality is one of the nine Bradford Hill criteria for causal inference in epidemiological studies, these criteria are used to evaluate whether there is sufficient human epidemiological evidence to infer a causal relationship [59, 60]. Although longitudinal studies alone would be insufficient to establish causality, it would contribute to the body of evidence towards establishing causality as one of the Bradford Hill criteria. This is significant since “[p]roof of a causal relationship would be a powerful weapon in the arsenal of both justice and health advocates” [2].

There are general and specific limitations regarding the exposure and outcome measurements. Generally, both the perceptions of the justice system and health outcomes were self-reported which may be subject to recall bias and response bias [61]. Furthermore, common method bias may be present since both were measured in the same survey using the same response method (ordinal scales) [62]. However, this may have been partially mitigated from the use of different scale formats (four-point and five-point Likert scales) and positively and negatively worded questions in layman language [62].

A specific limitation on measuring ATJ as exposure relate to the reliance on self-reported perceptions rather than *administrative data* (such as total case numbers and time required to resolve particular legal problems) which are generated by courts and justice sector institutions [11]. However, the literature suggests that this is not a glaring methodological issue since, such self-reports are good indicators for ATJ and can flag problems and populations that warrant investigation and intervention [12]. This is because perceptions of ATJ do not develop in a vacuum but rely on the actual circumstances [63, 64]. Furthermore, “people-centered” data is needed to meaningfully measure ATJ and can capture people’s experience of accessing justice through informal mechanisms which administrative data are blind to [53], indeed, administrative data are said to provide only “a narrow perspective of access to justice” [11]. This approach is recognised in the literature [14, 65], and by the Department of Justice of the Hong Kong Special Administrative Region (DOJ) which previously investigated the public’s perceived access to justice of the justice system as a whole (i.e., criminal and civil) [66]. Additionally, perceived ATJ plays a key role in shaping the realities of the legal system via “perception driving reality” [67]. Nonetheless, subjective measures of ATJ does not supplant administrative data and future studies should consider using both complementarily via a “triangulated” research design [11].

Furthermore, the present study was conducted after the 2019–2020 Hong Kong protests, the legal problems that arise surrounding the events primarily relate to the *criminal* justice system (such as the crime of unlawful assembly, rioting, perverting the course of justice, sedition, and other crimes relating to national security) which has received much media attention and has a number of ongoing cases to this day. It is worth repeating here that the present study is squarely concerned with perceived access to the *civil* justice system as opposed to the *criminal* justice system. Yet, as aforementioned, perceptions of access to *civil* justice do not exist in a vacuum but are influenced by surrounding circumstances [63, 64]. It is therefore submitted that how the Hong Kong government, courts, and wider public, react to the 2019–2020 protests and the resulting *criminal* cases will have a spill-over effect on influencing perceived accessibility to the *civil* justice system. Although the spill-over effect may be somewhat mitigated by a prompt in the survey explaining and providing examples of *civil* legal problems, the present study was unable to investigate such a spill-over effect, hence future research should explore this further and contribute towards developing a more accurate tool for measuring perceived access to the *civil* and *criminal* justice systems as separate constructs.

Another specific limitation of measuring perceived ATJ as exposure relate to the dimensionality of the modified IOJ-PIJ scale. As discussed in the prior validation study for the Traditional Chinese versions of the IOJ and PIJ, the modified IOJ-PIJ overemphasises the equality components of ATJ under the labels “procedural fairness” and “outcome neutrality”, other dimensions of ATJ have not been explored [27].

Specific limitations on measuring health outcomes using self-report scales include the difficulty in comprehensively and accurately assessing health status. However, it was methodologically sound to use validated scales especially under the time and resource constraints, notwithstanding that each of the validated scales had their own strengths and limitations. To address this limitation, future studies may consider obtaining medical records or other means to move away from self-reported health outcomes.

## Conclusion

ATJ is increasingly being considered as a key SDOH. This study provides empirical evidence that ATJ is a SDOH for specific dimensions of health. Overall, this study demonstrates that improving ATJ plays an important role in maintaining and improving population health and wellbeing. Hence, laws, policies, and initiatives to improve ATJ are encouraged. Consistent with the notion that assuring public health and wellbeing entails the collective efforts of society, the results of this study emphasise the mandate held by the legal system and its various actors within, along with the health sector through health-justice partnerships, and the broader community, to assure the public’s health by improving ATJ – improving ATJ is nothing less than a public health imperative.

### Electronic supplementary material

Below is the link to the electronic supplementary material.

Supplementary Material 1

Supplementary Material 2

Supplementary Material 3

## Data Availability

The datasets generated and analysed in this study are available as Supplementary Materials.

## Abbreviations

95% CI: 95% confidence interval
Anx: Anxiety
B: Unstandardised parameter estimates
CFI: Comparative fit index
Dep: Depression
DOJ: Department of Justice of the Hong Kong Special Administrative Region
DWLS: Diagonally weighted least square
Env: Environmental
GAD-2: Two-item generalised anxiety disorder scale
HM: Hypothetical model
IOJ: Inaccessibility of Justice scale
OECD: Organisation for Economic Co-operation and Development
Out.Neut: Outcome neutrality
PHQ-2: Two-item patient health questionnaire
PHQ-4: Four-Item Patient Health Questionnaire
Phy: Physical health
PIJ: Perceived Inequality of Justice scale
PRC: The People’s Republic of China
Pro.Fair: Procedural fairness
Psy: Psychological
RMSEA: Root mean square error of approximation
SCQ: Sangha’s Self-Administered Comorbidity Questionnaire
SDOH: Social determinant of health
SE B: Standard error of unstandardised estimates
SEM: Structural equation modelling
Soc: Social relationships
SRMR: Standardised root mean square residual
TLI: Tucker-Lewis index
WHO: World Health Organisation
WHOQOL-BREF(HK): Hong Kong version of the Abbreviated World Health Organization Quality of Life questionnaire
WJP: World Justice Project
β: Standardised parameter estimates
