# Supplementary material for: Access to civil justice as a social determinant of health: a legal epidemiological cross-sectional study
Source: Int J Equity Health. 2024 Jun 14;23:123. doi: 10.1186/s12939-024-02205-4 (PMC11179223; doi:10.1186/s12939-024-02205-4)
Supplement: Supplementary file 1 — Supplementary Material 1 [file 12939_2024_2205_MOESM1_ESM.pdf]

「訴諸司法」及「司法平等」 作為健康社會的決定因素：法律流行病學橫斷式研究

Access to Justice and Equality of Justice as Social Determinants of Health: A Legal Epidemiological Cross-sectional Study

參加者同意書

Informed Consent Form for Participants

1 **研究目的：**本研究旨在了解「訴諸民事司法」及「民事司法平等」與健康狀況的關係。
Aim of the study: This research survey aims to understand the relationship between 'access to civil justice' and 'equality of civil justice' with some health outcomes.

2 **參加者標準：**如果你年滿 18 歲，並居住於香港，就符合標準參加本研究。
Eligibility criteria: You are eligible to participate if you are at least 18 years old and living in Hong Kong.

3 **研究所需時間和項目：**完成填寫此問卷大約只需 20 分鐘。你會被問及一些有關你個人背景的問題（例如年齡、性別、教育背景等），你的健康狀況，以及你對香港「民事」司法制度的看法。
Duration and overview of the survey: It should take around 20 minutes to complete this survey. You will be asked some general questions about yourself (eg. age, sex, education background, etc.), your health, and your perceptions on the 'civil' justice system in Hong Kong.

4 **保密性：**是次研究調查中收集到有關你所有的資料及數據將被嚴格保密，並僅用於本研究目的。本問卷調查中收集的所有數據將在研究結束後的七年內被完全銷毀。
Confidentiality: All data collected about you in this survey will be kept strictly confidential and used for the research purposes of this study only. All data collected in this survey will be destroyed completely within seven years upon the completion of the study.

5 **研究補償：**如果你是首 100 名有效完成問卷調查，你可獲得 200 港元作為研究補償。在問卷調查結束時，會有一條問題詢問你的聯繫信息，研究人員會與你聯繫，詢問你能如何獲得研究補償。
Reimbursements: If you are the first 100 participants to validly complete the survey, you are eligible to receive HKD200 as reimbursement for your participation in the study. A question at the end of the survey will ask for your contact information and a researcher will contact you to ask how you would like to receive the reimbursement if you are eligible.

6 **退出研究的權益：**你以自願形式參與是次研究。參與這項研究不會給你帶來任何個人利益。你可以拒絕參與研究，甚至在調查進行中退出。你拒絕或退出研究的決定不會導致任何不良後果。
Right to withdraw: Your participation is completely voluntary. Participating in this research will not bring you any personal benefits. You may refuse to participate in the research and withdraw from the survey even while it is in progress. Your decision to refuse or withdraw from the study will not lead to any adverse consequences.

7 **聯絡資訊：**如對本研究有任何疑問，可透過電郵（[redacted]）或電話（[redacted]）與 [redacted] 的 [redacted]聯絡。作為研究參與者，如你覺得在是次調查期間的權利受到侵犯，你可以透過電子郵件（[redacted]）聯絡 *[redacted]*。

Contact information: If you have any questions about the study, you may contact **[redacted]** from the [redacted] by email **([redacted])** or by telephone **([redacted])**. If you feel your rights as a research participant has been violated during this survey, you may contact the [redacted] by email **([redacted])**.

8 **心理輔導：**如果你在研究期間的任何時候因研究而感到心理壓力，你可以通過電話（**852 2389 2222**）聯繫 *香港撒瑪利亞防止自殺會*，以獲得他們的 24 小時情緒支援熱線，或者通過電話（**852 2896 0000**）聯繫 *撒瑪利亞會* 的 24 小時中文及多種語言防止自殺熱線。
Psychological counselling: If you have experienced psychological stress due to the research at any time during the research, you can contact *The Samaritan Befrienders Hong Kong* by telephone (**852 2389 2222**) to access their 24-hour phone counselling services, or *The Samaritans* by telephone (**852 2896 0000**) to access their 24-hour multi-lingual suicide prevention hotline.

9 **同意聲明：**如果您瞭解上述資訊並願意參與本研究，請在以下橫線上簽署，填寫日期，然後繼續研究。
Consent statement: If you understand the above information and are willing to participate in this research, please sign on the line below, fill in the date, and continue to the survey.

參加者簽署 Signature of participant

日期 Date

請通過掃描 QR 碼以填寫電子版本的問卷，或填寫隨信附上的紙質版本，**不需要同時填寫兩份問卷**。
PLEASE COMPLETE EITHER THE ONLINE VERSION BY SCANNING THE QR CODE OR THE PAPER-BASED VERSION OF THE SURVEY, **NOT BOTH**.

請在 ☐ 內填 ✓ ，如 ☑。請回答所有問題。 **Please answer in the ☐ with a ✓, for example, ☑. Please answer all questions.**

甲部 PART A　「民事」司法制度的看法 PERCEPTIONS ON THE 'CIVIL' JUSTICE SYSTEM

本研究不涉及「刑事」司法系統。本研究涉及「民事」司法系統，該系統涉及如被僱主無理解雇、因他人疏忽而受傷、離婚涉及金錢糾紛或面臨被驅逐出家門等問題。思考民事司法系統中的問題，**您在多大程度上同意或不同意以下陳述。**

This study is NOT concerned with the 'criminal' justice system. This study is concerned with the 'civil' justice system that deals with issues such as being unreasonably sacked by your employer, injured as a result of someone else’s negligence, involved in a dispute over money as part of a divorce, or facing eviction from your home. Thinking about the issues in the civil justice system, **to what extent do you agree or disagree with the following statements.**

	非常不同意 **Strongly disagree**	不同意 **Mainly disagree**	同意 **Mainly agree**	非常同意 **Strongly agree**
這樣的問題通常會得到迅速有效的解決。<br>Issues like these are usually resolved promptly and efficiently.	☐	☐	☐	☐
不富裕人仕通常會得到不好的結果。<br>People with less money generally get a worse outcome.	☐	☐	☐	☐
法律就像一個遊戲，在這個遊戲中，熟練和足智多謀的人更有可能得到他們想要的東西。<br>For issues like these, law is like a game in which the skilful and resourceful are more likely to get what they want.	☐	☐	☐	☐
如有需要，很容易將此類問題告上法庭。<br>It is easy to take issues like these to court if needed.	☐	☐	☐	☐
對於這樣的問題，律師對於大多數人來說太昂貴。<br>For issues like these, lawyers are too expensive for most people to use.	☐	☐	☐	☐
司法系統物有所值。<br>The justice system provides good value for money.	☐	☐	☐	☐
對於這樣的問題，我可以負擔得起律師的幫助。<br>For issues like these, people like me can afford help from a lawyer.	☐	☐	☐	☐
富裕人仕的律師並不比貧窮人仕的律師好。<br>Rich people’s lawyers are no better than poor people’s lawyers.	☐	☐	☐	☐
將此類案件提交法庭通常比案件的價值損失更大。<br>Taking a case to court is generally more trouble than it is worth.	☐	☐	☐	☐
法律總是公平對待雙方，不論其背景、性別、種族或信仰。<br>The law always treat both parties fairly, whatever their background, gender, ethnicity or faith.	☐	☐	☐	☐
法官有自己的意圖，與法律分開。<br>Judges have their own agendas separate from the law.	☐	☐	☐	☐
法院的裁決和行動受到新聞界和政客壓力的影響。<br>The decisions and actions of courts are influenced by pressure from the press and politicians.	☐	☐	☐	☐
法院和法庭總是公平對待雙方，不論其背景、性別、種族或信仰。<br>Courts and tribunals always treat both parties fairly, whatever their background, gender, ethnicity or faith.	☐	☐	☐	☐

請在 ☐ 內填 ✓ ，如 ☑。請回答所有問題。 **Please answer in the ☐ with a ✓, for example, ☑. Please answer all questions.**

乙部 PART B　　健康狀況 HEALTH STATUS

在<u>過去兩個星期</u>，你有多經常受以下問題困擾? **Over the <u>last two weeks</u>, how often have you been bothered by the following problems?**

	完全沒有 Not at all	幾天 Several days	一半以上的天數 More than half the days	近乎每天 Nearly every day
感到緊張、不安或煩躁<br>Feeling nervous, anxious, or on edge	☐	☐	☐	☐
無法停止或控制憂慮<br>Not being able to stop or control worrying	☐	☐	☐	☐
情緒低落、抑鬱或絕望<br>Feeling down, depressed or hopeless	☐	☐	☐	☐
做任何事都覺得沉悶或者根本不想做任何事<br>Little interest of pleasure in doing things	☐	☐	☐	☐

以下落黎既題目，係問你<u>最近兩個星期</u>對於生活各方面的評價及睇法。每一個問題都係好重要，請你盡量回答所有問題。
The questions below ask you about certain things that happened in the <u>last two weeks</u> and your feelings about them.

你的主觀生活質素好唔好？How would you rate your quality of life?

☐ 極不好 Very poor　☐ 不好 Poor　☐ 無話好唔好 Neither poor nor good　☐ 好 Good　☐ 極好 Very good

你滿唔滿意你的健康狀況？How satisfied are you with your health?

☐ 極不滿意 Very dissatisfied　☐ 不滿意 Dissatisfied　☐ 無話滿唔滿意 Neither satisfied nor dissatisfied　☐ 好滿意 Satisfied　☐ 極滿意 Very satisfied

你有沒有時常覺得唔開心，例如情緒低落、絕望、焦慮、憂心、抑鬱等？
How often do you have negative feelings, such as blue mood, despair, anxiety, depression?

☐ 從來無 Never　☐ 好少有 Seldom　☐ 有時有 Quite often　☐ 好多時有 Very often　☐ 不停有 Always

你能唔能夠自己四圍去？How well are you able to get around?

☐ 不能夠 Very poor　☐ 少少能夠 Poor　☐ 某程度能夠 Neither poor nor well　☐ 好能夠 Well　☐ 完全能夠 Very well

你覺得其他人接唔接受你？(例如其他人會唔會當你係朋友，或會唔會討厭你、排斥你)
Do you think people are accepting of you? (For example, will other people treat you as their friend, or will they hate you, or reject you)

☐ 不接受 Not accepting　☐ 少少接受 A little accepting　☐ 某程度接受 Moderately accepting　☐ 好大程度接受 Very accepting　☐ 極接受 Extremely accepting

你容唔容易食到你想食的食物？How easy is it for you to eat the food you want to eat?

☐ 不容易 Not easy　☐ 少少容易 A little easy　☐ 某程度容易 Moderately easy　☐ 好大程度容易 Very easy　☐ 極容易 Extremely easy

	從來無 Not at all	少少 A little	有時有 A moderate amount	好大程度有 Very much	極有 An extreme amount
你覺唔覺得痛楚和唔舒服阻礙你做事？<br>To what extent do you feel that physical pain prevents you from doing what you need to do?	☐	☐	☐	☐	☐
你需唔需要靠醫療的幫助來應付日常生活？<br>How much do you need any medical treatment to function in your daily life?	☐	☐	☐	☐	☐
你享唔享受生活？<br>How much do you enjoy life?	☐	☐	☐	☐	☐
你覺得自己的生活有沒有意義？<br>To what extent do you feel your life to be meaningful?	☐	☐	☐	☐	☐

請在 ☐ 內填 ✓ ，如 ☑。請回答所有問題。 **Please answer in the** ☐ **with a** ✓, for example, ☑. **Please answer all questions.**

	從來無<br>**Not at all**	少少<br>**Slightly**	有時有<br>**A moderate amount**	好大程度有<br>**Very much**	極有<br>**Extremely**
你可唔可以集中精神？<br>How well are you able to concentrate?	☐	☐	☐	☐	☐
在日常生活中，你覺得安唔安全？<br>How safe do you feel in your daily life?	☐	☐	☐	☐	☐
你覺得你居住的區域的環境健唔健康?<br>How healthy is your physical environment?	☐	☐	☐	☐	☐

	不能夠<br>**Not at all**	少少能夠<br>**A little**	某程度能夠<br>**Moderately**	好能夠<br>**Mostly**	完全能夠<br>**Completely**
你能唔能夠有足夠精神來應付日常生活？<br>Do you have enough energy for everyday life?	☐	☐	☐	☐	☐
你能唔能夠接受自己的外貌？<br>Are you able to accept your bodily appearance?	☐	☐	☐	☐	☐
你能唔能夠有足夠的金錢應付需要？<br>Have you enough money to meet your needs?	☐	☐	☐	☐	☐
你能唔能夠得到你日常所需要的資訊？<br>How available to you is the information that you need in your day-to-day life?	☐	☐	☐	☐	☐
你能唔能夠有機會參加一些消遣活動？<br>To what extent do you have the opportunity for leisure activities?	☐	☐	☐	☐	☐

	極不滿意<br>**Very dissatisfied**	不滿意<br>**Dissatisfied**	無話滿唔滿意<br>**Neither satisfied nor dissatisfied**	好滿意<br>**Satisfied**	極滿意<br>**Very satisfied**
你睡得好唔好，滿唔滿意？<br>How satisfied are you with your sleep?	☐	☐	☐	☐	☐
你滿唔滿意自己做日常的事的能力？<br>How satisfied are you with your ability to perform your daily living activities?	☐	☐	☐	☐	☐
你滿唔滿意自己的工作能力？<br>How satisfied are you with your capacity for work?	☐	☐	☐	☐	☐
你滿唔滿意自己？<br>How satisfied are you with yourself?	☐	☐	☐	☐	☐
你滿唔滿意自己的人際關係？<br>How satisfied are you with your personal relationships?	☐	☐	☐	☐	☐
你滿唔滿意自己的性生活？<br>How satisfied are you with your sex life?	☐	☐	☐	☐	☐
你滿唔滿意朋友給你的支持？<br>How satisfied are you with the support you get from your friends?	☐	☐	☐	☐	☐
你滿唔滿意你現在居住的地方？<br>How satisfied are you with the conditions of your living place?	☐	☐	☐	☐	☐
你滿唔滿意現在醫療衛生服務的方便程度?<br>How satisfied are you with your access to health services?	☐	☐	☐	☐	☐
你滿唔滿意你用的交通工具？<br>How satisfied are you with your mode of transportation?	☐	☐	☐	☐	☐

以下是常見問題的清單。

第一列中，請指出您當前是否有問題。如果您沒有問題，請跳到下一個問題。

如果您有該問題，請在第二列中註明您是否接受了藥物或其他類型的治療，並在第三列中，指出問題是否限制了您的活動。

最後，在頁面末尾註明沒有列於「其他醫療問題」內的所有醫療狀況。

The following is a list of common problems.

In the first column, please indicate if you currently have the problem. If you do not have the problem, skip to the next problem.

If you do have the problem, please indicate in the second column if you receive medications or some other type of treatment for the problem; and in the third column, indicate if the problem limits any of your activities.

Finally, indicate all medical conditions that are not listed under "other medical problems" at the end of the page.

		你有這樣的問題嗎？ Do you have the problem?		你有接受治療嗎？ Do you receive treatment for it?		它會限制您的活動嗎？ Does it limit your activities?	
問題	Problem	沒有 No	有 Yes	沒有 No	有 Yes	沒有 No	有 Yes
心臟病	Heart disease	☐	☐ →	☐	☐	☐	☐
高血壓	High blood pressure	☐	☐ →	☐	☐	☐	☐
肺病	Lung disease	☐	☐ →	☐	☐	☐	☐
糖尿病	Diabetes	☐	☐ →	☐	☐	☐	☐
潰瘍或胃病	Ulcer or stomach disease	☐	☐ →	☐	☐	☐	☐
腎病	Kidney disease	☐	☐ →	☐	☐	☐	☐
肝病	Liver disease	☐	☐ →	☐	☐	☐	☐
貧血或其他血液疾病	Anemia or other blood disease	☐	☐ →	☐	☐	☐	☐
癌症	Cancer	☐	☐ →	☐	☐	☐	☐
抑鬱症	Depression	☐	☐ →	☐	☐	☐	☐
骨關節炎、退行性關節炎	Osteoarthritis, degenerative arthritis	☐	☐ →	☐	☐	☐	☐
背痛	Back pain	☐	☐ →	☐	☐	☐	☐
類風濕性關節炎	Rheumatoid arthritis	☐	☐ →	☐	☐	☐	☐
其他醫療問題（請填寫）Other medical problems (please write in)							
請在這空間填寫	WRITE IN THIS SPACE	☐	☐ →	☐	☐	☐	☐
請在這空間填寫	WRITE IN THIS SPACE	☐	☐ →	☐	☐	☐	☐

請在 ☐ 內填 ✓ ，如 ☑。請回答所有問題。 **Please answer in the ☐ with a ✓, for example, ☑. Please answer all questions.**

丙部 PART C 人口信息 DEMOGRAPHIC INFORMATION

您居住於香港哪個區?
Which district do you live in?

☐ 中西區 Central & Western ☐ 九龍城 Kowloon City ☐ 元朗 Yuen Long
☐ 灣仔 Wan Chai ☐ 黃大仙 Wong Tai Sin ☐ 北區 North
☐ 東區 Eastern ☐ 觀塘 Kwun Tong ☐ 大埔 Tai Po
☐ 南區 Southern ☐ 葵青 Kwai Tsing ☐ 沙田 Sha Tin
☐ 油尖旺 Yau Tsim Mong ☐ 荃灣 Tsuen Wan ☐ 西貢 Sai Kung
☐ 深水埗 Sham Shui Po ☐ 屯門 Tuen Mun ☐ 離島 Islands

您的年齡是？
What is your age?

☐ 15 – 19 ☐ 20 – 24 ☐ 25 – 29 ☐ 30 – 34 ☐ 35 – 39
☐ 40 – 44 ☐ 45 – 49 ☐ 50 – 54 ☐ 55 – 59 ☐ 60 – 64
☐ 65 – 69 ☐ 70 – 74 ☐ 75 – 79 ☐ 80 – 84 ☐ 85 或以上 85 or older

您的性別是？
What is your sex?

☐ 男 Male ☐ 女 Female ☐ 非二元性別 / 第三性別 Non-binary / third gender
☐ 其他（請註明）Other (please specify) ______________________________
☐ 不願意說明 Prefer not to say

您的性取向是什麼？
What is your sexual orientation?

☐ 異性戀 Heterosexual or straight ☐ 同性戀 Homosexual ☐ 雙性戀 Bisexual
☐ 其他（請註明）Other (please specify) ______________________________
☐ 不想透露 Prefer not to say

您的婚姻狀況如何？
What is your marital status?

☐ 未結婚或單身 Not yet married or single ☐ 已婚 Married
☐ 同居 Living as married or cohabitate ☐ 喪偶 Widowed
☐ 離婚 Divorced ☐ 分居 Separated

您的就業狀況如何？(可選多於一項)
What is your employment status? (You may choose more than one answer)

☐ 全職 Employed full-time ☐ 全職自僱 Self-employed full-time
☐ 兼職 Employed part-time ☐ 兼職自僱 Self-employed part-time
☐ 業主 Business owner ☐ 家務主夫/家庭主婦 Homemaker/domestic duties
☐ 待業（求職中）Unemployed looking for work
☐ 待業（不在求職中）Unemployed not looking for work
☐ 退休 Retired ☐ 學生 Student ☐ 残疾 Disabled
☐ 其他（請註明）Other (please specify) ______________________________

請問您的母語是什麼？ (可選多於一項)
Which is/are your native language? (You may choose more than one answer)

☐ 廣東話 Cantonese ☐ 普通話 Putonghua ☐ 英文 English
☐ 其他（請註明）Other (please specify) ______________________________

哪裡是你的出生地？
What is your birthplace?

☐ 香港特別行政區 Hong Kong SAR, China
☐ 香港特別行政區以外 Outside Hong Kong SAR, China

請在 ☐ 內填 ✓ ，如 ☑。請回答所有問題。 **Please answer in the ☐ with a ✓, for example, ☑. Please answer all questions.**

您的最高教育水平是什麼？<br>**What is your highest attained education level?**	☐ 沒有正規教育 No formal education ☐ 小學或以下 Primary or below<br>☐ 初中（中一至中三）Lower secondary (forms 1-3)<br>☐ 高中（中四至六或中七）Upper secondary (forms 4-6 or 7)<br>☐ 副學士學位或更高文憑 Associate degree or higher diploma<br>☐ 本科或學士學位 Undergraduate degree or Bachelor degree<br>☐ 碩士學位 Master's degree ☐ 博士 Doctorate
您的<u>家庭</u>月收入水準是多少？（以港幣計算）<br>**What is your monthly <u>household</u> income level? ($ in HKD)**	☐ 少於 $10,000 Less than $10,000 ☐ $10,000 - $19,999<br>☐ $20,000 - $29,999 ☐ $30,000 - $39,999 ☐ $40,000 - $49,999<br>☐ $50,000 - $59,999 ☐ $60,000 - $69,999 ☐ $70,000 - $79,999<br>☐ $80,000 - $89,999 ☐ $90,000 - $99,999 ☐ $100,000 - $149,999<br>☐ 多於 $150,000 More than $150,000
請問您的家中共有幾人同住？（包括您自己在內）<br>**What is your household size (meaning the number of people living under one roof)?**	☐ 1 ☐ 2 ☐ 3 ☐ 4 ☐ 5<br>☐ 6 ☐ 7 ☐ 8 ☐ 9 ☐ 10 或以上 10 or more
你有香港永久性居民身份嗎？<br>**Do you have Hong Kong permanent resident status?**	☐ 有 Yes ☐ 沒有 No ☐ 不清楚 Don't know
請問您屬於哪個族裔？ **(可選多於一項)**<br>**What is your ethnicity? (You may choose more than one answer)**	☐ 華人 Chinese ☐ 菲律賓人 Filipino ☐ 印尼人 Indonesian<br>☐ 白人 White ☐ 日本人 Japanese ☐ 尼泊爾人 Nepalese<br>☐ 巴基斯坦人 Pakistani ☐ 泰國人 Thai ☐ 印度人 Indian<br>☐ 其他（請註明）Others (please specify) ______________________

丁部 **PART D** 補償 **REIMBURSEMENT**

在這項研究中，如果你是首 **100** 名有效完成调查的參加者，你可獲得 **200** 港元作為研究補償。請提供你的聯繫信息，研究人員將與你聯繫，安排補償事宜。請確保您提供的聯繫信息是準確的。 **(你可在以下提供一種或兩種聯絡方法)**

In this study, if you are the first 100 participants to validly complete the survey, you are eligible to receive HKD200 as reimbursement for your participation in the study. Please provide your contact information and a researcher will contact you to arrange the reimbursement. Please ensure the contact information that you provide is accurate. (You may provide one or both contact methods below)

電話號碼（可用於 WhatsApp）
Telephone number (can be reached by WhatsApp) ______________________

電子郵件
Email address ______________________

***如果你選擇不提供聯任何聯絡信息，研究人員將無法聯繫到你來安排補償事宜，即使你可獲得研究補償，你也不會得到補償。**

***If you choose not to provide any contact information, the researcher will not be able to contact you to arrange the reimbursement and you will not get the reimbursement even if you are eligible.**

-問卷調查結束
我們希望您為這次經歷感到有意義。
感謝您的參與！

This is the end of the survey
We hope you have found the experience to be meaningful.
Thank you for your participation!
