# Supplementary material for: Access to civil justice as a social determinant of health: a legal epidemiological cross-sectional study
Source: Int J Equity Health. 2024 Jun 14;23:123. doi: 10.1186/s12939-024-02205-4 (PMC11179223; doi:10.1186/s12939-024-02205-4)
Supplement: Supplementary file 3 — Supplementary Material 3 [file 12939_2024_2205_MOESM3_ESM.docx]

Table A: Structural model and parameter estimates for HM1

| Structural model for HM1 | | B (95% CI) | β (95% CI) | SE B | p-value |
| --- | --- | --- | --- | --- | --- |
| Latent variables: |  |  |  |  |  |
| Procedural fairness =~ | IOJ1 | 1 | 0.332 (0.286 - 0.378) |  |  |
|  | IOJ6 | 1.633 (1.374 - 1.892) | 0.555 (0.502 - 0.609) | 0.132 | <0.05* |
|  | IOJ7 | 1.214 (1.007 - 1.421) | 0.431 (0.381 - 0.481) | 0.106 | <0.05* |
|  | IOJ8 | 1.207 (0.991 - 1.424) | 0.359 (0.314 - 0.404) | 0.110 | <0.05* |
|  | PIJ1 | 2.496 (2.120 - 2.871) | 0.768 (0.706 - 0.829) | 0.192 | <0.05* |
|  | PIJ4 | 2.338 (1.984 - 2.692) | 0.780 (0.717 - 0.843) | 0.181 | <0.05* |
| Outcome neutrality =~ | IOJ2 | 1 | 0.787 (0.720 - 0.853) |  |  |
|  | IOJ3 | 0.703 (0.619 - 0.787) | 0.598 (0.539 - 0.656) | 0.043 | <0.05* |
|  | IOJ5 | 0.472 (0.409 - 0.535) | 0.435 (0.385 - 0.486) | 0.032 | <0.05* |
|  | IOJ9 | 0.460 (0.394 - 0.527) | 0.406 (0.354 - 0.459) | 0.034 | <0.05* |
|  | PIJ2 | 0.210 (0.151 - 0.269) | 0.177 (0.129 - 0.225) | 0.030 | <0.05* |
|  | PIJ3 | 0.620 (0.536 - 0.705) | 0.455 (0.403 - 0.508) | 0.043 | <0.05* |
| Physical health =~ | WHOQOL_BREF_HK4 | 1 | 0.475 (0.440 - 0.510) |  |  |
|  | WHOQOL_BREF_HK7 | 0.668 (0.605 - 0.731) | 0.320 (0.292 - 0.348) | 0.032 | <0.05* |
|  | WHOQOL_BREF_HK8 | 0.625 (0.568 - 0.683) | 0.325 (0.295 - 0.355) | 0.029 | <0.05* |
|  | WHOQOL_BREF_HK14 | 1.405 (1.314 - 1.496) | 0.764 (0.719 - 0.810) | 0.047 | <0.05* |
|  | WHOQOL_BREF_HK19 | 1.225 (1.142 - 1.308) | 0.632 (0.595 - 0.669) | 0.042 | <0.05* |
|  | WHOQOL_BREF_HK20 | 1.235 (1.153 - 1.316) | 0.753 (0.704 - 0.801) | 0.042 | <0.05* |
|  | WHOQOL_BREF_HK21 | 1.162 (1.083 - 1.240) | 0.689 (0.645 - 0.734) | 0.040 | <0.05* |
| Psychological =~ | WHOQOL_BREF_HK3 | 1 | 0.582 (0.544 - 0.619) |  |  |
|  | WHOQOL_BREF_HK5 | 1.105 (1.039 - 1.172) | 0.652 (0.608 - 0.697) | 0.034 | <0.05* |
|  | WHOQOL_BREF_HK6 | 1.199 (1.127 - 1.271) | 0.628 (0.586 - 0.669) | 0.037 | <0.05* |
|  | WHOQOL_BREF_HK9 | 1.477 (1.395 - 1.560) | 0.759 (0.717 - 0.801) | 0.042 | <0.05* |
|  | WHOQOL_BREF_HK10 | 1.500 (1.414 - 1.587) | 0.683 (0.645 - 0.721) | 0.044 | <0.05* |
|  | WHOQOL_BREF_HK11 | 1.105 (1.040 - 1.170) | 0.649 (0.609 - 0.689) | 0.033 | <0.05* |
|  | WHOQOL_BREF_HK15 | 1.489 (1.404 - 1.574) | 0.711 (0.670 - 0.752) | 0.043 | <0.05* |
|  | WHOQOL_BREF_HK22 | 1.275 (1.201 - 1.349) | 0.724 (0.680 - 0.768) | 0.038 | <0.05* |
| Social relationships =~ | WHOQOL_BREF_HK23 | 1 | 0.797 (0.738 - 0.856) |  |  |
|  | WHOQOL_BREF_HK24 | 0.716 (0.669 - 0.762) | 0.587 (0.538 - 0.635) | 0.024 | <0.05* |
|  | WHOQOL_BREF_HK25 | 0.721 (0.676 - 0.765) | 0.660 (0.606 - 0.714) | 0.023 | <0.05* |
| Environmental =~ | WHOQOL_BREF_HK12 | 1 | 0.640 (0.598 - 0.682) |  |  |
|  | WHOQOL_BREF_HK13 | 0.886 (0.829 - 0.943) | 0.553 (0.516 - 0.590) | 0.029 | <0.05* |
|  | WHOQOL_BREF_HK16 | 1.179 (1.111 - 1.248) | 0.695 (0.655 - 0.735) | 0.035 | <0.05* |
|  | WHOQOL_BREF_HK17 | 0.983 (0.923 - 1.043) | 0.652 (0.609 - 0.694) | 0.030 | <0.05* |
|  | WHOQOL_BREF_HK18 | 1.164 (1.096 - 1.232) | 0.708 (0.667 - 0.749) | 0.034 | <0.05* |
|  | WHOQOL_BREF_HK26 | 0.871 (0.815 - 0.928) | 0.561 (0.524 - 0.598) | 0.029 | <0.05* |
|  | WHOQOL_BREF_HK27 | 0.882 (0.825 - 0.940) | 0.541 (0.506 - 0.576) | 0.029 | <0.05* |
|  | WHOQOL_BREF_HK28 | 0.839 (0.784 - 0.893) | 0.558 (0.520 - 0.597) | 0.028 | <0.05* |
| Regressions: |  |  |  |  |  |
| Physical health ~ | Procedural fairness | -0.325 (-0.437 - -0.212) | -0.153 (-0.201 - -0.105) | 0.058 | <0.05* |
|  | Outcome neutrality | -0.067 (-0.114 - -0.019) | -0.071 (-0.121 - -0.021) | 0.024 | <0.05* |
| Psychological ~ | Procedural fairness | -0.326 (-0.421 - -0.232) | -0.162 (-0.203 - -0.121) | 0.048 | <0.05* |
|  | Outcome neutrality | -0.119 (-0.158 - -0.079) | -0.133 (-0.176 - -0.091) | 0.020 | <0.05* |
| Social relationships ~ | Procedural fairness | -0.680 (-0.896 - -0.465) | -0.228 (-0.293 - -0.162) | 0.110 | <0.05* |
|  | Outcome neutrality | -0.021 (-0.110 - 0.067) | -0.016 (-0.084 - 0.051) | 0.045 | 0.638 |
| Environmental ~ | Procedural fairness | -0.535 (-0.671 - -0.398) | -0.211 (-0.255 - -0.166) | 0.070 | <0.05* |
|  | Outcome neutrality | -0.137 (-0.191 - -0.083) | -0.122 (-0.169 - -0.076) | 0.028 | <0.05* |
| Covariances: |  |  |  |  |  |
| Procedural fairness ~~ | Outcome neutrality | 0.064 (0.053 - 0.074) | 0.486 (0.439 - 0.533) | 0.005 | <0.05* |
| Physical health ~~ | .Psychological | 0.218 (0.202 - 0.234) | 0.929 (0.891 - 0.968) | 0.008 | <0.05* |
|  | .Social relationships | 0.266 (0.244 - 0.288) | 0.761 (0.699 - 0.823) | 0.011 | <0.05* |
|  | .Environmental | 0.240 (0.221 - 0.258) | 0.817 (0.778 - 0.856) | 0.009 | <0.05* |
| .Psychological ~~ | .Social relationships | 0.265 (0.247 - 0.283) | 0.812 (0.754 - 0.869) | 0.009 | <0.05* |
|  | .Environmental | 0.232 (0.217 - 0.247) | 0.849 (0.816 - 0.882) | 0.008 | <0.05* |
| .Social relationships ~~ | .Environmental | 0.276 (0.254 - 0.297) | 0.676 (0.622 - 0.731) | 0.011 | <0.05* |
| *p<0.05, **p<0.01, ***p<0.001; "." before the variable name indicates the residual of the variable. | | | |  |  |

Table B: Structural model and parameter estimates for HM2

| Structural model for HM2 | | B (95% CI) | β (95% CI) | SE B | p-value |
| --- | --- | --- | --- | --- | --- |
| Latent Variables: |  |  |  |  |  |
| Procedural fairness =~ | IOJ1 | 1 | 0.368 (0.317 - 0.419) |  |  |
|  | IOJ6 | 1.450 (1.206 - 1.693) | 0.547 (0.488 - 0.605) | 0.124 | <0.05* |
|  | IOJ7 | 0.961 (0.779 - 1.143) | 0.378 (0.325 - 0.431) | 0.093 | <0.05* |
|  | IOJ8 | 1.122 (0.912 - 1.332) | 0.370 (0.320 - 0.420) | 0.107 | <0.05* |
|  | PIJ1 | 2.311 (1.945 - 2.677) | 0.788 (0.719 - 0.857) | 0.187 | <0.05* |
|  | PIJ4 | 2.109 (1.771 - 2.446) | 0.779 (0.710 - 0.849) | 0.172 | <0.05* |
| Outcome neutrality =~ | IOJ2 | 1 | 0.716 (0.647 - 0.784) |  |  |
|  | IOJ3 | 0.844 (0.730 - 0.958) | 0.652 (0.586 - 0.718) | 0.058 | <0.05* |
|  | IOJ5 | 0.556 (0.475 - 0.638) | 0.466 (0.410 - 0.522) | 0.042 | <0.05* |
|  | IOJ9 | 0.527 (0.443 - 0.611) | 0.423 (0.366 - 0.480) | 0.043 | <0.05* |
|  | PIJ2 | 0.192 (0.122 - 0.262) | 0.147 (0.095 - 0.199) | 0.036 | <0.05* |
|  | PIJ3 | 0.674 (0.570 - 0.779) | 0.450 (0.393 - 0.507) | 0.053 | <0.05* |
| Anxiety =~ | PHQ4_GAD2_1 | 1 | 0.849 (0.757 - 0.940) |  |  |
|  | PHQ4_GAD2_2 | 1.043 (0.900 - 1.187) | 0.874 (0.780 - 0.969) | 0.073 | <0.05* |
| Depression =~ | PHQ4_PHQ2_1 | 1 | 0.846 (0.746 - 0.946) |  |  |
|  | PHQ4_PHQ2_2 | 0.988 (0.852 - 1.124) | 0.756 (0.668 - 0.844) | 0.069 | <0.05* |
| Regressions: |  |  |  |  |  |
| Anxiety ~ | Procedural fairness | 0.335 (0.172 - 0.498) | 0.132 (0.070 - 0.193) | 0.083 | <0.05* |
|  | Outcome neutrality | 0.091 (0.003 - 0.179) | 0.066 (0.002 - 0.130) | 0.045 | <0.05* |
| Depression ~ | Procedural fairness | 0.313 (0.148 - 0.478) | 0.130 (0.064 - 0.197) | 0.084 | <0.05* |
|  | Outcome neutrality | 0.115 (0.025 - 0.204) | 0.089 (0.020 - 0.157) | 0.046 | <0.05* |
| Covariances: |  |  |  |  |  |
| Procedural fairness ~~ | Outcome neutrality | 0.064 (0.053 - 0.075) | 0.486 (0.439 - 0.533) | 0.006 | <0.05* |
| .Anxiety ~~ | .Depression | 0.391 (0.343 - 0.439) | 0.930 (0.809 - 1.050) | 0.024 | <0.05* |
| *p<0.05, **p<0.01, ***p<0.001; "." before the variable name indicates the residual of the variable. | | | |  |  |

Table C: Structural model and parameter estimates for HM3

| Structural model for HM3 | | B (95% CI) | β (95% CI) | SE B | p-value |
| --- | --- | --- | --- | --- | --- |
| Latent Variables: |  |  |  |  |  |
| Procedural fairness =~ | IOJ1 | 1 | 0.376 (0.324 - 0.429) |  |  |
|  | IOJ6 | 1.435 (1.192 - 1.677) | 0.553 (0.493 - 0.614) | 0.124 | <0.05* |
|  | IOJ7 | 0.920 (0.742 - 1.097) | 0.370 (0.317 - 0.424) | 0.091 | <0.05* |
|  | IOJ8 | 1.069 (0.864 - 1.273) | 0.360 (0.310 - 0.411) | 0.104 | <0.05* |
|  | PIJ1 | 2.266 (1.903 - 2.628) | 0.790 (0.719 - 0.861) | 0.185 | <0.05* |
|  | PIJ4 | 2.060 (1.727 - 2.394) | 0.779 (0.708 - 0.850) | 0.170 | <0.05* |
| Outcome neutrality =~ | IOJ2 | 1 | 0.694 (0.626 - 0.762) |  |  |
|  | IOJ3 | 0.870 (0.749 - 0.991) | 0.652 (0.585 - 0.719) | 0.062 | <0.05* |
|  | IOJ5 | 0.592 (0.504 - 0.680) | 0.481 (0.424 - 0.539) | 0.045 | <0.05* |
|  | IOJ9 | 0.551 (0.461 - 0.640) | 0.429 (0.370 - 0.487) | 0.046 | <0.05* |
|  | PIJ2 | 0.219 (0.145 - 0.293) | 0.163 (0.110 - 0.216) | 0.038 | <0.05* |
|  | PIJ3 | 0.701 (0.591 - 0.812) | 0.454 (0.396 - 0.512) | 0.057 | <0.05* |
| Regressions: |  |  |  |  |  |
| Comorbidity ~ | Procedural fairness | 1.369 (0.490 - 2.248) | 0.126 (0.044 - 0.208) | 0.448 | <0.05* |
|  | Outcome neutrality | -0.801 (-1.298 - -0.303) | -0.130 (-0.213 - -0.047) | 0.254 | <0.05* |
| Covariances: |  |  |  |  |  |
| Procedural fairness ~~ | Outcome neutrality | 0.063 (0.052 - 0.074) | 0.484 (0.437 - 0.531) | 0.006 | <0.05* |
| *p<0.05, **p<0.01, ***p<0.001; "." before the variable name indicates the residual of the variable. | | | |  |  |

Table D: Structural model and parameter estimates for HM3 age-adjusted

| Age-adjusted structural model for HM3 | | B (95% CI) | β (95% CI) | SE B | p-value |
| --- | --- | --- | --- | --- | --- |
| Latent Variables: |  |  |  |  |  |
| Procedural fairness =~ | IOJ1 | 1 | 0.373 (0.321 - 0.426) |  |  |
|  | IOJ6 | 1.442 (1.197 - 1.687) | 0.552 (0.492 - 0.612) | 0.125 | <0.05* |
|  | IOJ7 | 0.925 (0.745 - 1.104) | 0.369 (0.316 - 0.423) | 0.092 | <0.05* |
|  | IOJ8 | 1.084 (0.876 - 1.291) | 0.362 (0.312 - 0.413) | 0.106 | <0.05* |
|  | PIJ1 | 2.289 (1.922 - 2.657) | 0.792 (0.721 - 0.863) | 0.188 | <0.05* |
|  | PIJ4 | 2.080 (1.742 - 2.418) | 0.780 (0.709 - 0.851) | 0.172 | <0.05* |
| Outcome neutrality =~ | IOJ2 | 1 | 0.701 (0.634 - 0.768) |  |  |
|  | IOJ3 | 0.852 (0.737 - 0.967) | 0.645 (0.579 - 0.711) | 0.059 | <0.05* |
|  | IOJ5 | 0.580 (0.495 - 0.664) | 0.476 (0.420 - 0.532) | 0.043 | <0.05* |
|  | IOJ9 | 0.530 (0.445 - 0.615) | 0.417 (0.360 - 0.474) | 0.043 | <0.05* |
|  | PIJ2 | 0.211 (0.139 - 0.283) | 0.158 (0.106 - 0.211) | 0.037 | <0.05* |
|  | PIJ3 | 0.717 (0.608 - 0.826) | 0.468 (0.411 - 0.526) | 0.056 | <0.05* |
| Regressions: |  |  |  |  |  |
| Comorbidity ~ | Procedural fairness | 0.975 (0.026 - 1.924) | 0.089 (0.002 - 0.176) | 0.484 | <0.05* |
|  | Outcome neutrality | -0.059 (-0.648 - 0.530) | -0.010 (-0.106 - 0.087) | 0.300 | 0.844 |
|  | Age | 0.409 (0.293 - 0.524) | 0.431 (0.288 - 0.574) | 0.059 | <0.05* |
| Procedural fairness ~ | Age | -0.004 (-0.009 - 0) | -0.049 (-0.098 - -0.001) | 0.002 | <0.05* |
| Outcome neutrality ~ | Age | -0.037 (-0.047 - -0.028) | -0.240 (-0.294 - -0.186) | 0.005 | <0.05* |
| Covariances: |  |  |  |  |  |
| .Procedural fairness ~~ | .Outcome neutrality | 0.062 (0.051 - 0.073) | 0.488 (0.438 - 0.538) | 0.006 | <0.05* |
| *p<0.05, **p<0.01, ***p<0.001; "." before the variable name indicates the residual of the variable. | | | |  |  |
